# Supplementary figures and images for: Effects of temperature on metabolic scaling in black carp
Source: PeerJ. 2020 May 27;8:e9242. doi: 10.7717/peerj.9242 (PMC7261118; doi:10.7717/peerj.9242)

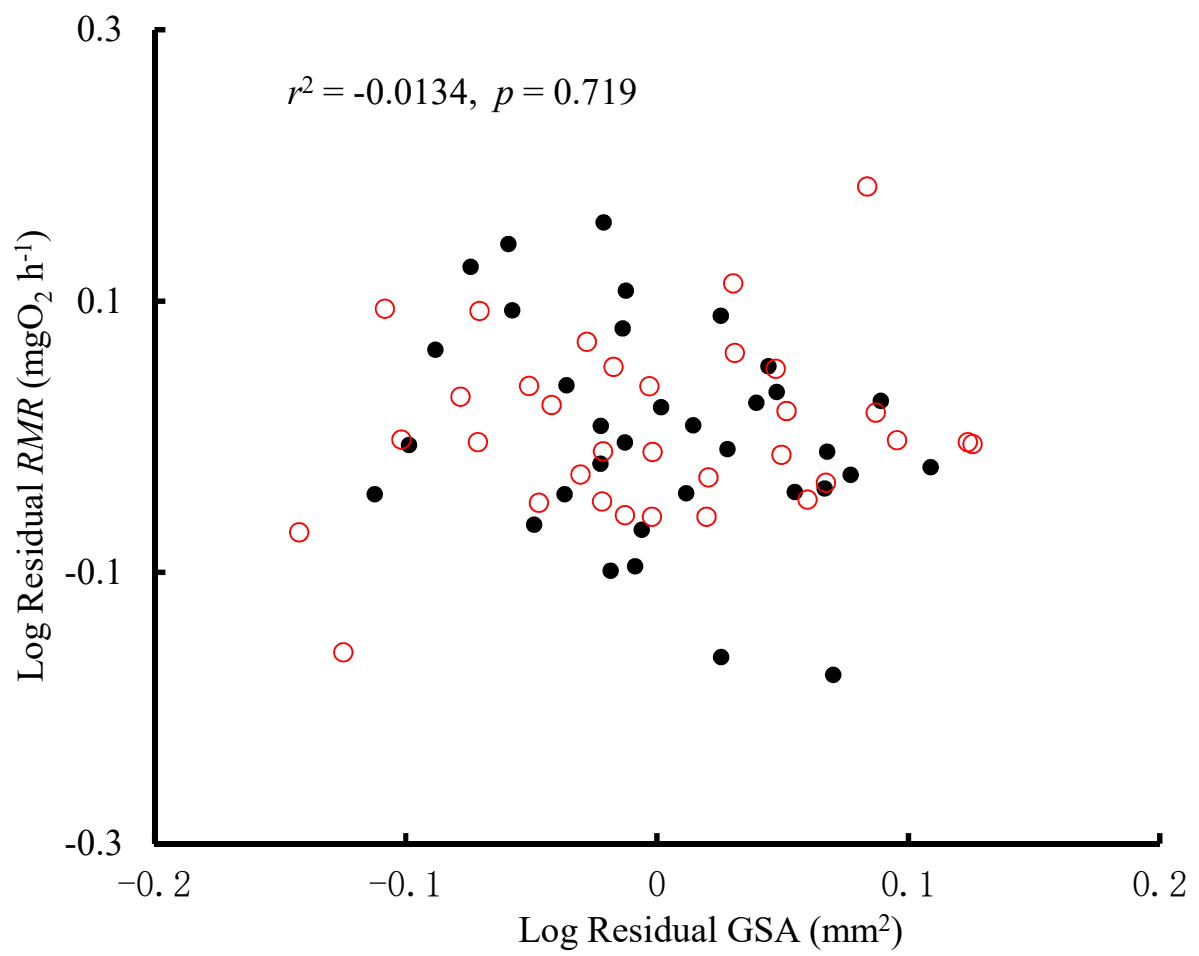

Supplement: Supplemental Information 1 — Correlation between the residual gill surface area (GSA, mm2) and the residual resting metabolic rate (RMR, mg O2 h−1) of the black carp Mylopharyngodon piceus treated by two temperatures (T, °C) for 2 weeks. No significant effects of both M and T on GSA was observed using the general linear model (glm). No significant correlation between GSA and M was observed using the Pearson’s correlation analyses. red open circles: 25 °C; black filled circles: 10 °C. [file peerj-08-9242-s001.pdf]

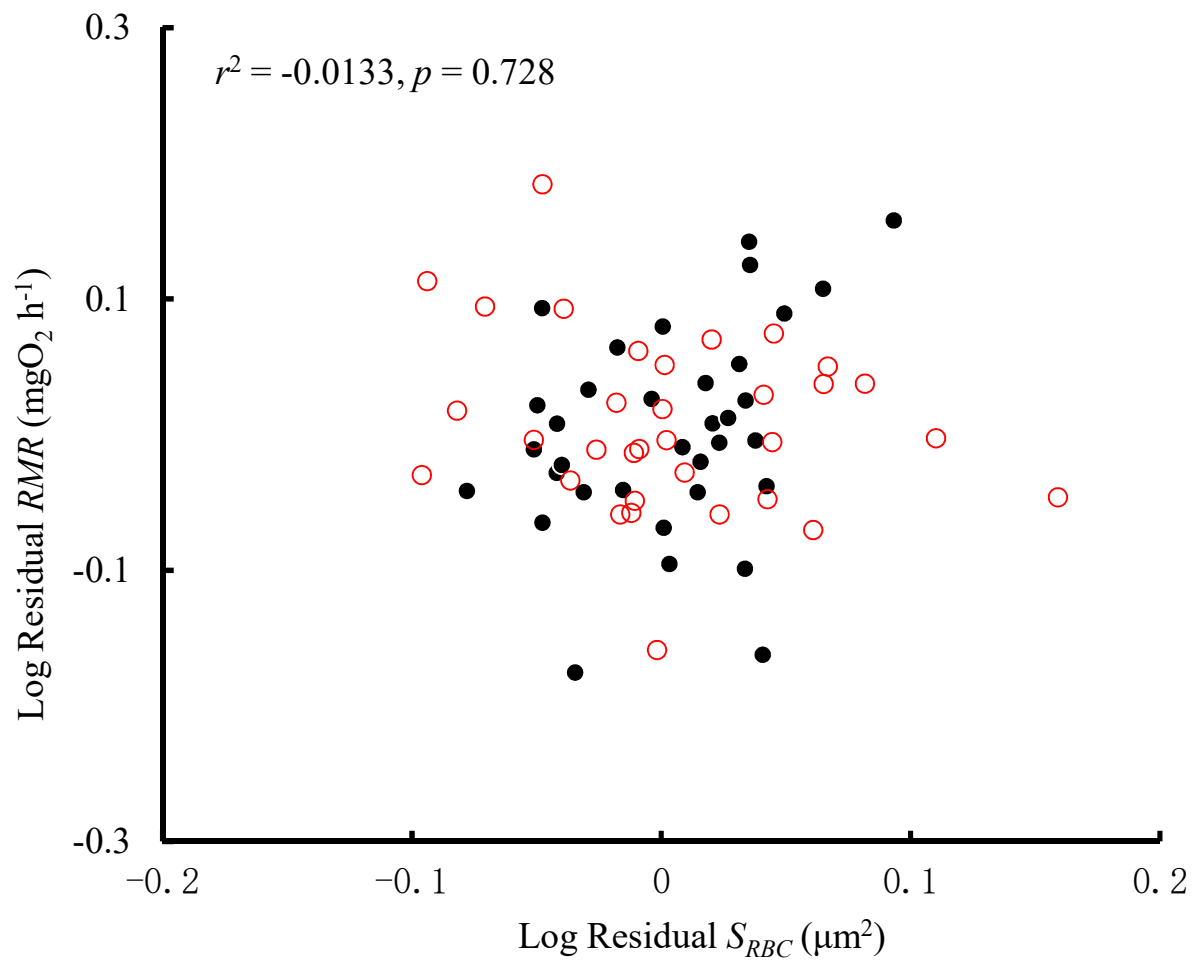

Supplement: Supplemental Information 2 — Correlation between the residual red blood cell size (SRBC, μm2) and the residual resting metabolic rate (RMR, mg O2 h−1) of the black carp Mylopharyngodon piceus treated by two temperatures (T, °C) for 2 weeks. No significant effects of both M and T on SRBC was observed using the general linear model (glm). No significant correlation between SRBC and M was observed using the Pearson’s correlation analyses. red open circles: 25 °C; black filled circles: 10 °C. [file peerj-08-9242-s002.pdf]
